# Critical Analysis of Neuronal Cell and the Mouse Bioassay for Detection of Botulinum Neurotoxins

**DOI:** 10.3390/toxins11120713

**Published:** 2019-12-07

**Authors:** Sabine Pellett, William H. Tepp, Eric A. Johnson

**Affiliations:** Department of Bacteriology, University of Wisconsin-Madison, 1550 Linden Dr, Madison, WI 53706, USA; sabine.pellett@wisc.edu (S.P.); whtepp@wisc.edu (W.H.T.)

**Keywords:** botulinum neurotoxin, BoNT, detection, mouse bioassay, cell assay, neurons, in vitro BoNT assays

## Abstract

Botulinum Neurotoxins (BoNTs) are a large protein family that includes the most potent neurotoxins known to humankind. BoNTs delivered locally in humans at low doses are widely used pharmaceuticals. Reliable and quantitative detection of BoNTs is of paramount importance for the clinical diagnosis of botulism, basic research, drug development, potency determination, and detection in clinical, environmental, and food samples. Ideally, a definitive assay for BoNT should reflect the activity of each of the four steps in nerve intoxication. The in vivo mouse bioassay (MBA) is the ‘gold standard’ for the detection of BoNTs. The MBA is sensitive, robust, semi-quantitative, and reliable within its sensitivity limits. Potential drawbacks with the MBA include assay-to-assay potency variations, especially between laboratories, and false positives or negatives. These limitations can be largely avoided by careful planning and performance. Another detection method that has gained importance in recent years for research and potency determination of pharmaceutical BoNTs is cell-based assays, as these assays can be highly sensitive, quantitative, human-specific, and detect fully functional holotoxins at physiologically relevant concentrations. A myriad of other in vitro BoNT detection methods exist. This review focuses on critical factors and assay limitations of the mouse bioassay and cell-based assays for BoNT detection.

## 1. Introduction

Botulinum neurotoxins (BoNTs) are potent neurotoxins causing serious and potentially fatal human and vertebrate animal disease botulism. BoNTs are also important and widely used pharmaceuticals. Used in small doses and applied locally by intramuscular injection, BoNTs can provide long-lasting relief to many neurologic disorders, including dystonias, spasticity, pain, and many other applications, including cosmesis [1,2,3,4,5]. However, only 10-fold higher doses than the highest-used therapeutic doses are estimated to be lethal to humans if injected intravenously. This is due to the more rapid systemic distribution of BoNTs after intravenous administration compared to intramuscular injection. Once in the circulatory system, BoNTs distribute to the peripheral nervous system where they enter neuronal cells, with preferential entry into motor-neurons. Inside a neuron, the toxins block neurotransmitter release, leading to the long-lasting descending bilateral paralysis characteristic of botulism [6]. Even if applied intramuscularly for therapeutic purposes, a portion of the injected BoNTs, in particular at high doses, can diffuse away from the local injection site leading to distal neuronal effects and at very high doses systemic distribution [7,8,9,10]. BoNTs can also enter human or vertebrate circulation and cause botulism by different routes, including ingestion of contaminated foods, by wound infection with neurotoxigenic clostridia, and by the colonization of the intestinal tract by neurotoxigenic clostridia, causing infant botulism or adult intestinal botulism [11,12]. The latter is rare, as *C. botulinum* usually does not colonize a healthy intestine with a mature microbiota. The severe symptoms of botulism last from several days to up to a year, depending on the BoNT type and dose. Without treatment, botulism has a mortality rate of up to 50%, but with supportive treatment as well as early antitoxin administration, mortality is reduced to below 5% [13].

Due to the high potency of BoNTs, with low nanogram quantities being lethal if injected intravenously, and low microgram quantities being lethal by oral consumption [6], combined with the lack of antidotes and required intensive medical care, BoNTs are considered potential bioweapons and are classified as Tier 1 Select Agents (www.selectagents.gov). In addition, BoNTs are a perennial concern in the food industry, and continued food safety testing is of paramount importance to prevent foodborne botulism outbreaks. BoNTs also have been exceedingly useful tools in neuroscience research due to their ability to completely block neurotransmitter release by neurons, affecting many physiological processes. Finally, the large family of BoNTs continues to expand with the discovery of new subtypes and chimeric toxins, and much remains to be learned about the biology and evolutionary significance of these novel toxins [14,15,16,17]. Detection of BoNTs is essential for all facets of BoNTs, including safety, the morbidity of botulism, regulatory decisions and actions, pharmaceutical development, and use of BoNTs, and basic research. This review discusses considerations for the detection of BoNTs, with the main emphasis on the detection of biologically active BoNTs by the classic mouse bioassay and by cell-based assays.

## 2. The Botulinum Neurotoxins Family

BoNTs comprise a large family of naturally occurring neurotoxins. BoNTs are 150 kDa AB-type endopeptidases consisting of a heavy chain (HC) (~100 kDa) and a light chain (LC) (~ 50 kDa) linked by a disulfide bond [18,19]. The LC is a zinc-dependent endopeptidase that cleaves and inactivates neuronal SNARE (soluble N-ethylmaleimide-sensitive factor activating protein receptor) proteins [20]. SNARE proteins are an essential component of the neuronal transmitter release machinery [21,22], and their cleavage by BoNTs disrupts this functionality, leading to a block in neurotransmitter release. The HC is structurally and functionally subdivided into the C-terminal receptor-binding domain (H_CC_) and the N-terminal translocation domain (H_CN_) [18]. The H_CC_ facilitates specific neuronal cell binding and entry via ganglioside and protein receptor binding, leading to endocytosis of the entire BoNT molecule [18,23,24,25]. Inside the endosome, structural changes occur, leading to the insertion of the H_CN_ domain into the endosome membrane and translocation of the LC into the cell cytosol. Inside the cell cytosol, the disulfide bond between HC and LC is cleaved, and the catalytically active LC is released into the cell cytosol [26,27,28]. BoNTs predominantly affect motor-neurons of the peripheral nervous system, thus leading to a block in neuronal activation of muscles resulting in flaccid paralysis. Thus, neuronal cell entry consists of four steps and requires all three functional domains of BoNTs for (1). neuronal cell binding, (2). endocytosis, (3). translocation of the LC into the neuronal cell cytosol, and (4). enzymatic activity of the LC inside the neuronal cell cytosol.

**Figure 1:**
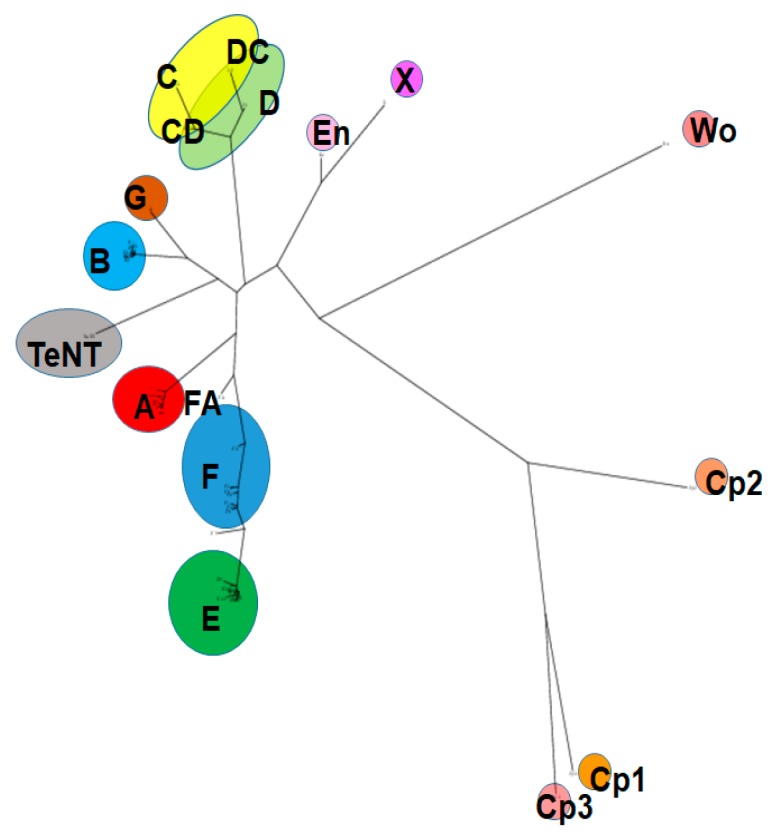
BoNT (botulinum neurotoxin) and homologs protein family. Amino acid sequences of published BoNTs and homologs were aligned in Clustal Omega. The phylogeny tree was created in PhyML using a minimum of SH-like and chi2-based-like likelihood ratio test, the Dayhoff substitution model, and was rendered using Drawtree.

Based on immunological differences, BoNTs have been classified into seven distinct serotypes (A–G) [29], and most of the serotypes are subdivided into subtypes based on differences in their amino acid sequence. Subtypes within one serotype are denoted by numbers after letters (i.e., BoNT/A1-9). Over 40 distinct BoNT subtypes, including some serotype chimeras, have been described, and new subtypes continue to be identified. Amino acid sequence variations between BoNT serotypes range from 35–70%, and between subtypes within one serotype from 2.6% to 32% [14,16,30,31]. A potential 8^th^ serotype was discovered recently by sequence analysis and named BoNT/X; however, it is currently unknown whether the BoNT-like molecule has biological activity, and recombinantly produced protein appears to be non-toxic or only mildly toxic to vertebrates [32]. BoNTs are produced primarily by the Gram-positive, anaerobic, spore-forming bacterial species *Clostridium botulinum* [31,33,34,35]. In addition, genetically related homologs of BoNTs have also been identified in more distantly related genera, including *Enterococcus*, *Weissella*, and *Chryseobacterium* [17,36,37,38,39,40,41,42]. However, while the catalytic activity of these BoNT-like LCs on SNARE proteins has been shown, there are currently no indications that these homologs act as vertebrate neurotoxins, and further research will be required to determine potential toxicity, target hosts, and biologic function. Figure 1 shows a phylogeny tree of published BoNT sequences and more distantly related homologs.

The various serotypes and subtypes of BoNTs differ in key structural and functional sites in the receptor and ganglioside binding domains as well as the catalytic domains, defining the serotype and subtype-specific cell entry and catalytic characteristics [15,16,43,44]. Most BoNT subtypes have not been isolated and functionally and structurally analyzed, but much has been learned about the subtype 1 of the various serotypes. BoNT serotypes A, D, E, and F bind to SV2 receptors as their protein receptor with various affinities and SV2 isotype specificities [45,46,47,48], whereas BoNT/B and G bind to synaptotagmin I and II with different affinity and specificity [49,50,51]. In addition, ganglioside binding specificity and affinity varies amongst the serotypes. BoNT/B and/C bind gangliosides with greater affinity than BoNT/A1 [52], while BoNT/A2 appears to interact with polysialogangliosides with greater affinity than BoNT/A1 [53,54]. Cell entry kinetics also differ within the BoNT types, with BoNT/E,/A2, and/A6 entering neuronal cells faster than BoNT/A1 [55,56,57]. BoNT sero-and subtypes also differ in catalytic activity and intracellular persistence, with BoNT/A1 and/C1 having the longest reported intracellular duration of action, followed by BoNT/B and/D, then BoNT/A3, BoNT/E and/F [56,58,59,60,61,62]. Each BoNT serotype cleaves SNARE substrates at a distinct cleavage site. BoNT/A, C, and E each cleave SNAP-25 at unique sites [63,64]. BoNT/C also cleaves syntaxin [65,66,67]. BoNT/B, D, G, and F each cleave synaptobrevin-1 and 2 (VAMP1 and 2) at specific sites [68,69,70,71]. The subtypes within each serotype cleave SNAREs at the same unique cleavage sites of each serotype, yet substrate recognition sequences and enzyme kinetics can vary, and much remains to be learned. Interestingly, recent research of novel BoNTs has found that BoNT/F5 and BoNT/FA are an exception within the F subtype LCs in that they cleave VAMP1/2 (vesicle-associated membrane protein) at unique sites, which are distinct from the cleavage sites of other type F LCs [72,73]. Interestingly, the novel putative BoNT/X, as well as BoNT homologs from organisms other than clostridia such as BoNT/Wo and BoNT/En, have been found to have unique neuronal and in some cases non-neuronal SNARE cleavage targets and sites [17,32,36,37,38,39,40,41,42]. It is currently unknown whether novel or as yet uncharacterized BoNTs may have unique SNARE cleavage targets, which is an important consideration when using a BoNT detection method that relies on detection of specific SNARE cleavage.

## 3. Considerations for Botulinum Neurotoxin Detection Methods

Reliable quantitative and qualitative detection of BoNTs is the basis for all research and botulism prevention efforts as well as clinical treatment of patients for neurological disorders and cosmesis. Historically, BoNTs have been detected over many decades by the mouse bioassay (MBA), in which mice are injected with BoNTs using an appropriate stabilization buffer, and surviving or symptom-free mice are counted [74,75,76,77]. The MBA still is the primary method for the detection and quantification of BoNTs. In efforts to avoid animal suffering, a multitude of BoNT detection methods has been developed with minimal use of animals. These include in vivo simulation assays, such as the hemidiaphragm assay and local injection assays [57,59,62,78,79,80,81,82], numerous in vitro assays using immunological detection methods, endopeptidase assays, or a combination of the two such as the endopeptidase-mass spectrometry assay [83,84,85,86,87,88], and cell-based assays [89].

Certain of these assays are equally or more sensitive than the MBA. However, most in vitro assays have specific characteristics that restrict their utility. Immunological detection methods such as ELISA detect BoNTs by specific antibody binding and thereby are restricted by antibody specificity to validated subtypes, and most immunological assays do not differentiate between biologically active and inactive or partially degraded BoNTs. Endopeptidase assays detect the proteolytic activity of LCs, usually by cleavage of synthetic substrates comprising a portion of the SNAREs, or by cleavage of recombinant SNARE motifs in vitro. Thereby these assays require only the presence of the enzymatic activity rather than the actions of fully functional holotoxin. Thus, they are substrate-dependent, and may not equally detect all subtypes within one serotype or a novel BoNT with a novel SNARE substrate or cleavage site. Even assays combining immunologic detection and endopeptidase activity are limited by not measuring each step in the four-step intoxication pathway. These assays can be reliably used for the detection of the BoNT isotypes for which they have been optimized and validated only [90]. When selecting a BoNT detection assay, sero- and subtype-specific differences and whether there is a need to detect all sero- or sub-types of BoNTs must be considered. Validations of in vitro assays for all subtypes are hampered by the regulatory and technical challenges of producing purified BoNTs of all subtypes, most of which have not yet been isolated and characterized [14]. Despite the restrictions for in vitro assays, they have the advantage that controls, and toxin standards can readily be included, and that many of the in vitro assays detect BoNTs rapidly and generally at low costs. These in vitro assays can be excellent choices for specific, well-defined applications that do not require determination of the biologic activity of holotoxins, including monitoring commercial BoNT production or rapid detection in clinical or food samples. While careful sample preparation methods and selection of assay materials and parameters can overcome some of the limitations of in vitro BoNT detection assays, only a biologic assay such as the MBA or an appropriate cell-based assay can selectively detect biologically active BoNTs, as well as all subtypes and novel BoNTs.

When selecting a BoNT detection assay, it is important to consider the end goal, such as laboratory diagnosis of botulism, detection in food or environmental samples, regulatory actions, pharmaceutical activity and properties, and toxicity evaluations of novel BoNTs [83,91].

Applications for BoNT detection include fundamental research, investigations of environmental samples or food products for epidemiologic investigations, food safety laboratory studies, analysis of clinical samples, quantification of pharmacologic BoNTs, and diagnosis of botulism. Each application has specific needs and requirements [83]. For food safety studies, the presence of biologically active or inactive BoNTs would indicate potential contamination of the food. Thus, the relatively high sensitivity and lower cost of an immunologic detection method may be of advantage for many food safety studies. However, the dependence of this method on specific antibody recognition raises the potential for false negatives due to lower affinity or failed antibody binding of a specific or novel subtype in samples that may contain unknown BoNTs, and for false positives due to non-specific recognition.

For analysis of clinical specimens and epidemiologic analysis of environmental or food samples, speed and high sensitivity are important factors. There currently is no BoNT detection method that is ideally suited for this purpose. The MBA is a robust and reliable assay that detects all BoNTs, but it may take up to four days or longer for completion depending on the toxin type, although the results are often available in one day. The MBA can lack the necessary sensitivity to detect the low levels of BoNTs often found in clinical samples from botulism patients, with data indicating that the MBA was negative in 30–40% of positively confirmed botulism cases [92,93,94,95]. Several in vitro assays with increased sensitivity [84,87,88], some of which are completed within hours, are useful for this application. However, these assays may not detect novel BoNTs and BoNTs for which the assays have not been validated, and require careful and thorough validation, ideally between independent laboratories. Several recent reviews provide detailed and comprehensive information on in vitro detection methods for BoNTs [83,84,87,88,91].

For the use of BoNT for research purposes, it is of particular importance to consider the biologic activities of BoNTs, especially when examining functional characteristics of novel BoNTs or for BoNTs that have not been purified to the 150 kDa neurotoxic form. For example, when molar equivalents of two different BoNTs are compared for potency, duration, and pharmacodynamics after local administration, while one BoNT preparation has 10-times the biologic activity of the other, results may lead to misleading conclusions about the two toxins. It should be noted that the specific activity of several laboratory preparations of the same BoNT type can vary significantly from one preparation to the next, and between laboratories. Such variations could be due to BoNT preparation and purification methods, BoNT purity, storage conditions, degree of enzymatic activation of the toxin (conversion to the more active di-chain form), stability and inactivation, and other factors. Comparative functional analyses of uncharacterized BoNTs to known BoNT types, therefore, must include specific toxicity determination in order that the same quantities of biologically active toxin can be functionally compared.

For potency (specific toxicity) determination of pharmaceutical BoNTs, where the quantitative and specific determination of biologically active BoNT of a specific and well-defined product is critical, an assay that considers all biologic steps of intoxication must be used. Both the mouse bioassay and cell-based assays fulfill this requirement and are being used for this purpose [74,89,96,97,98].

## 4. Neuronal Cell-Based Assays

### 4.1. Overview of Neuronal Cell-Based Assays

Neuronal cell-based assays (NCB assays) have been used for decades for BoNT research and have led to many important discoveries about these toxins. These assays expose a neuronal cell population to BoNT in defined media or buffer for a defined amount of time, followed by the analysis of an intracellular BoNT function such as SNARE protein cleavage or a block in neurotransmitter release or signal transduction. Comprehensive reviews on these methods are published elsewhere [89,99,100,101]. Historically, these assays have used primary neuronal cultures derived from rodents or chickens, as well as continuous cell lines derived from cancer cells. In the past decade, an explosion of advancements in tissue culture techniques and genetics, materials, and derivation and culture of stem cell-derived neurons have enabled the refinement and optimization of several NCB assays that can reliably and quantitatively detect BoNTs with greater sensitivity and less variation than the mouse bioassay [96,98,102,103,104,105,106,107,108,109,110].

NCB assays using human-induced pluripotent stem cell (hiPSC) derived neurons or modified cell lines are being used successfully and continue to be developed for potency determination of pharmaceutical BoNTs. Before the introduction and first approval of an NCB assay for potency determination of pharmaceutical BoNTs, the MBA was the only US Food and Drug Adminsitration (FDA) approved assay for this purpose in the US. This is due to the requirement of quantitative detection of biologically active BoNT for administration to humans, as the accurate dosing of bio-active pharmaceutical BoNTs is of paramount importance to achieve the desired and long-lasting effect while avoiding unwanted side effects, in particular, systemic distribution of the toxin. Only an assay that requires all steps of the cellular intoxication process, including cell surface binding, endocytosis, translocation of the light chain into the cellular cytosol, and enzymatic activity of the light chain on SNARE substrates can accurately determine biologically active BoNT. NCB assays currently are the only assays other than in vivo animal assays that fulfill this requirement. The introduction of a highly standardized, reproducible, and sensitive NCB assay now largely enables replacement of the MBA for this purpose, avoiding the use of hundreds of thousands of mice per year. Furthermore, the use of hiPSC derived neurons now provides a human-specific assay, overcoming the challenge of species specificity associated with animal models and opening the possibility of standardizing pharmaceutical BoNTs in terms of human specific activity. Currently, pharmaceutical BoNT/B1 preparations require about 40-fold higher mouse LD50 Units to achieve the same therapeutic effect as BoNT/A1 in humans due to human-specific lower activity of BoNT/B1, which binds the human protein receptor synaptotagmin II with lower affinity than mouse synaptotagmin II [2,111,112,113].

NCB assays are also much-used research models to study neuronal cell function, including cell entry mechanisms, the intracellular catalytic activity of the LC, duration of action, antibody neutralization, inhibitor screens and characterizations, and other aspects important for the use of BoNTs in humans and animal models. Neuronal cell models offer defined and species-specific models, which are particularly useful in investigating cell entry mechanisms, intracellular persistence, and properties of novel and as yet uncharacterized BoNTs.

Some of the same aspects that make NCB assays useful for certain research applications and potency determination of specific BoNT formulation also are important limitations for these and other applications. Cultured neuronal cells, irrespective of their source, always have cell-population specific characteristics that can affect the outcome of a BoNT assay. The cell source, culture substrate, media, culture conditions, feeding schedule, and any added factors such as serum or growth factors all can affect the outcome of a BoNT assay either directly or indirectly by affecting cell health. While many of these factors are controllable, each specific neuronal cell model has additional limitations. Some cell models lack sensitivity, whereas other cell models are mixed populations of neurons and glia, and other cell models are highly sensitive but either difficult to prepare or expensive to purchase. All cell models are species-specific for the species of origin, and all cell models require sterile samples for testing, are susceptible to matrix effects, and require at least two days for completion if maximum sensitivity is needed. These are major limitations for applications such as testing of environmental or food samples or for diagnostic testing. These limitations are discussed further in the following sections.

### 4.2. Types of Neuronal Cell-Based Assays

While NCB assays provide an excellent detection platform for quantitative and qualitative assessments of biologically active BoNTs, careful considerations must be given to the specific properties, advantages, and limitations of each NCB assay. ‘One hat does not fit all’ applies for NCB assays, as reviewed elsewhere [89]. NCB assays can be conducted using continuous cell lines, primary neuronal cell populations, or stem cell-derived neuronal cell populations. The culture methods used for each affect the composition and characteristics of the neuronal cells, which in turn affect BoNT sensitivity. Continuous cell lines, while renewable, inexpensive, and easy to use, generally lack sensitivity to BoNTs and often lack parts of the SNARE machinery or cell surface receptors. Some cell lines can be made more sensitive by chemical differentiation, but care must be taken to fully characterize the cells after differentiation, as their metabolism, ganglioside profile, and protein expression pattern may be altered. Neuronal cells differentiated from cell lines usually are viable for only a few days, and it is currently unknown how associated changes affect BoNT uptake and SNARE cleavage. On the other hand, continuous cell lines are often highly defined pure populations and can be more easily manipulated than primary or stem cell-derived neurons.

Primary neurons are relatively inexpensive and highly sensitive to BoNTs but are typically animal-derived and require the sacrifice of some animals, as well as skilled personnel for the preparation of cells and performance of the assay. Primary cells can be obtained from different central nervous system regions (e.g., spinal cord, dorsal root ganglion, hippocampal, or cortical neurons), from different species, and by various protocols including enrichment protocol for specific subsets of neuron types, all of which determine the specific characteristics of the resulting cell population. Primary cells constitute mixed cultures of various neuronal subclasses and supportive (glial) cells; however, several protocols have been established for consistent preparation and properties of specific and defined primary neuronal cell populations. Most primary neuronal cultures can be maintained in culture for several weeks to months if measures are taken to avoid overgrowth of glial cells, enabling their use in long-term studies.

Equally sensitive but more defined cell models are stem cell-derived neuronal cultures. Mouse or human stem cells are now routinely differentiated to various types of neuronal subpopulations, with achievable neuron purity of 90–98% and over 80% enrichment for specific neuron types. Since the advent of human-induced pluripotent stem cells (hiPSCs) [114,115,116], most efforts have focused on deriving neuronal subpopulations from this cell source, which does not require the use of any animals or embryonic tissue. The hiPSC derived neuronal cell models have the important advantage that they represent non-cancerous neuronal subpopulations containing fully functional endocytosis and exocytosis mechanisms, are human-specific, and can be maintained in culture for several weeks to months.

When designing an NCB assay for a specific purpose, it is important to carefully choose the appropriate cell model and consider the specific characteristics of each cell model when interpreting results. In addition, the culture methods, surface coating, media composition, and other factors can have dramatic and specific effects on neuronal cell health, morphology, and function [117]. Thus, when comparing a rodent and a human cell model for sensitivity to a specific BoNT, greater sensitivity in the human cell model does not necessarily imply greater sensitivity in vivo in humans, and mechanistic studies demonstrating human specificity would be required for that conclusion. In fact, a recent study has shown that BoNT sensitivity of various hiPSC derived neuronal cell models varies significantly for the same BoNT with motor-neurons being the most sensitive, and that the sensitivity of the same model varied significantly for various BoNT serotypes [110].

### 4.3. Critical Factors Affecting the Detection of BoNTs in Neuronal Cell-Based Assays

In addition to the selection of an appropriate cell model, many other factors affect the outcome of an NCB assay. All cell-based assays are comparative. In other words, the activity of a BoNT preparation can be compared to that of others or a BoNT standard in one cell model and using the same assay parameters, but another cell model or new cell preparation may yield different results. Another critical factor for a successful NCB assay is overall cell health. The variation in culture conditions, including temperature, pH variations, cell age, nutrient deficiency, cellular stress, poor surface attachment, or presence of cytotoxins, growth factor, proteins, excipients, salts, metals, sugars, or detergents can cause significant changes in cell physiology that may affect BoNT uptake, intracellular trafficking, SNARE cleavage, or neurotransmitter release. This can be largely optimized by the inclusion of appropriate controls and toxin standards, but careful consideration of these factors is required when interpreting results. For example, if a differentiated cell population is beginning to die, and the cell membrane integrity is compromised, BoNT may access the SNARE proteins in the cell population without specific cell entry. If a neuronal cell population is induced to undergo endocytosis, some BoNT may be taken up non-specifically, followed by unknown intracellular trafficking. This same phenomenon may occur at doses of BoNTs above the physiologically relevant range. In fact, research has shown non-specific uptake of BoNT fragments at concentrations above 10 nM [96], which is also corroborated by unpublished data from our laboratory. NCB assays should not rely on results of one BoNT concentration, in particular, if that concentration is in the nM range or greater. Assay specific artifacts that can easily be missed with one toxin concentration can be avoided by using a dose-range.

Another cell type and toxin type-specific factor impacting the BoNT sensitivity of NCB assays is the incubation time of cells with BoNT. Most cells require 24-72 h incubations for maximal sensitivity, but the toxin exposure time can be reduced to 2–10 min by chemically stimulating neuronal activity in cells with 56–80 mM KCl and 1–2 mM CaCl_2_ at 37 °C [55,56,98]. In this method, it is important to consider that several hours of incubation is required after toxin removal to achieve efficient intracellular SNARE cleavage. Typically, SNARE cleavage becomes detectable after 1–2 h, and continues to increase for 24–48 h, depending on toxin dose and type. It is important to keep in mind that this activity-dependent rapid BoNT uptake is highly dependent on the synaptic vesicle recycling activity of the cells, which is cell type-specific. Thus, if a particular NCB assay is used to determine an IC50 of an intracellular BoNT inhibitor, this IC50 may be higher or lower in another cell model.

Additional consideration must be given to the endpoint used to determine BoNT activity. In addition to some endpoints being more sensitive than others, there are critical factors specific for each endpoint. For example, factors that affect exocytosis, such as calcium concentration, salt concentrations, temperature, ion channel inhibitors or activators, as well as other factors must be considered when neurotransmitter release or signal transduction is used as an endpoint. If cells are pre-loaded with a dye to measure exocytosis via dye release, factors that may alter endocytosis also have to be controlled. Electric conductance measured by MEA (multi-electrode array) or patch-clamp is sensitive to many factors, including temperature, ion and salt concentrations, presence of inhibitors or inducers of signal transmission, technical specifications, etc. Direct measurement of SNARE cleavage by Western blot or ELISA relies on complete cell lysis and prevention of protein degradation, which may not equally affect cleaved and uncleaved SNARE fragments.

### 4.4. Critical Considerations for BoNT Detection by Cell-Based Assays

Cell-based assays are a ‘closed system’, and even though they can replace an in vivo assay for specific applications if carefully controlled and standardized, they cannot be used as a blanket replacement. This is particularly important in light of BoNTs being neurotoxins and the complexity and plasticity of the nervous system in vivo. NCB assays are excellent models for investigating specific BoNT cell entry characteristics in physiologically relevant models, enabling studies that would not be possible in vivo due to the many other factors involved in in vivo intoxication. However, NCB assays do not necessarily determine the in vivo potency of a novel BoNT, as they cannot consider the pharmacodynamic and pharmacokinetic properties that might play important roles in an in vivo assay. The pharmacodynamics of a compound given to an animal include absorption, distribution, metabolism, and elimination. Neuronal cell selectivity of BoNTs in vivo is guided by distribution, metabolism, and secretion [118], whereas in cultured neurons, only the assay parameters and cell-specific characteristics such as ganglioside profiles and neuronal cell surface receptor expression play a role. Studies have shown that after intravenous administration, BoNTs are rapidly (within a few minutes) distributed throughout the general circulation, where the toxins are stable for up to several days with a very slow elimination phase [119,120]. BoNTs must pass from the circulation into the extravascular compartment to access peripheral neurons.

Early studies using radiolabeled BoNT/A or/B exposure of in vitro vertebrate tissue samples indicated a preferential selectivity for cholinergic motor nerve ending and minimal binding to non-neuronal cells, but also binding to in vitro exposed brain section regions [121,122]. Cell-based assays have shown large variability in the sensitivity of various cell models, with primary neuronal cell models and stem cell-derived neurons being the most sensitive, and hiPSC-derived motor neurons being more sensitive for most BoNTs than other hiPSC-derived neuronal classes [89,110]. However, further studies examining the molecular mechanisms controlling potential neuronal cell selectivity by BoNTs are required to confirm the preferential uptake of BoNTs by cholinergic neurons, as the results of in vivo studies could be due to pharmacodynamic properties of the toxins, and the results of the NCB studies could be due to extracellular or cell model-specific factors other than their cholinergic properties. In addition, retrograde and anterograde transport of BoNTs can affect their observed pathology. For example, in NCB assays, the BoNT related tetanus toxin (TeNT) would appear to have a similar mode of action of entering neuronal cells and cleaving a SNARE protein, but in vivo it causes drastically different symptoms of spasticity versus the flaccid paralysis caused by BoNTs, because after uptake into peripheral motor-neurons it is retrogradely transported and transcytosed to the inhibitory neurons inside the spinal cord [123,124]. While NCB assays can be set up specifically to examine retrograde transport and transcytosis [125], in vivo animal assays are required to determine toxin specific pathology.

Potency determinations of different BoNTs by NCB assay and MBA do not necessarily correlate. Several BoNT types (A2, A6, FA) have been shown to enter cultured neurons more efficiently, but BoNT/FA was significantly less potent in mice [55,57,61,126,127]. Thus, greater potency of a BoNT in NCB assays doesn’t necessarily mean greater toxicity in vivo. For toxicity investigations of the many not yet isolated and characterized BoNT subtypes, as well as novel BoNTs or putative BoNTs, in vivo assays such as the MBA, therefore, remain important, while specific NCB assays are valuable by contributing cell entry and intracellular function data in an isolated system that can be defined with regards to species specificity, culture conditions, cell specific characteristics, etc.

There are several circumstances that require the detection of multiple BoNT types. These include analysis of environmental, clinical, or food samples, and laboratory investigations of novel or putative BoNTs. For these applications, an assay that can detect the biologic activity of multiple BoNT sero- and sub-types is required. While cell-based assays can provide human-specific and highly sensitive BoNT detection platforms fulfilling this requirement, research in the past few years has indicated that the activity of BoNTs in cultured neurons and in vivo in mice does not equally correlate for all BoNTs [55,57,61,126,128]. Thus, the mouse bioassay remains an important detection method for BoNTs, in particular, for investigations of novel BoNTs and for detection of BoNTs in complex matrices such as stool samples and food matrices.

## 5. The Mouse Bioassay

### 5.1. Overview of the Mouse Bioassay

The mouse bioassay has been the ‘gold standard’ assay for the detection of BoNTs for decades and for good reasons. It is reliable, sensitive, detects all known BoNTs, can be quantitative, detects only biologically active BoNT, and can be used to detect BoNTs from many matrices. In fact, BoNTs are measured in mouse LD50 Units, which are defined as the amount of BoNT required to kill 50% of mice within four days after intraperitoneal injection. The methods for the mouse bioassay is detailed in the Bacteriological Analytical Manual (BAM) [74,129]. The MBA is the only BoNT detection assay that considers all aspects of BoNT intoxication, including steps involved in neuronal cell entry, and also pharmacodynamics of the toxins. While the classic quantal MBA uses an intraperitoneal injection of BoNTs, which results in a rapid systemic distribution of the toxins, other routes of administration including oral, intravenous, subcutaneous, intramuscular, and inhalation can also be examined by lethal or non-lethal assays. Symptoms are seen most rapidly after intravenous administration, and time-to-death curves have been established for BoNT/A,/B, and/E [130,131]. While rapid, these time-to-death curves require high concentrations of toxin, they only provide semi-quantitative estimates, and are specific for the BoNT type and preparation method. While one study has indicated that the time-to-death for the same number of mouse LD50 Units of 900 kDa BoNT/A complex differs significantly from that for purified 150 kDa BoNT/A [132], another study showed no difference [130]. Variations for time-to-death by subtypes within one serotype have also been reported [61]. Intramuscular and subcutaneous injections, usually of sub-lethal doses, combined with assays assessing the resulting local paralysis, are usually used to study pathology, onset, and duration of action of sub-lethal doses of BoNTs. Significant differences between BoNT types have been demonstrated using such studies [57,58,59,62,126,133].

The most important drawback of the MBA is the ethical concerns with using large numbers of animals in an assay that is associated with distress for the animals. In addition, the MBA is expensive, requires approved animal facilities and staff, and carries a risk for staff as it requires handling the most potent toxins known to humankind with sharps (syringes). Accidents from sharps is the most common cause of laboratory accidents. While the MBA provides an in vivo system for BoNT detection, thereby enabling the detection of biologic activity and evaluating pharmacodynamic properties of the toxins, it is inherently species-specific for mice. The importance of this is demonstrated by the example of BoNT/B1, which has an equal specific activity to BoNT/A1 in mice, but about 40-fold lower activity in humans due to a species-specific difference in the neuronal cell protein receptor for BoNT/B1, synaptotagmin II [134,135]. Nevertheless, for most BoNTs, the mouse appears to be an excellent model to predict toxicity in humans, and it is the only assay that considers all aspects of cellular and in vivo intoxication.

### 5.2. Variability of Quantitative BoNT Detection by the Mouse Bioassay

As for any in vivo assay, the MBA has a significant standard error. In addition, the MBA is not standardized across laboratories, and considerable variations occur within and between laboratories, with an intra-laboratory error of up to 20% and inter-laboratory error of over 50%. The introduction of a toxin standard and determining a relative BoNT activity by the MBA has been shown to significantly reduce the error [136], and purified recombinantly produced toxin standards for BoNT/A-F have been proposed [137]. However, it is logistically challenging to introduce a universal BoNT standard to be used across laboratories worldwide and with various types and formulations of BoNTs. Since the activity of a toxin standard also needs to be determined by the MBA to be a relevant standard for active BoNT, such a standard would have to originate from one source and would need significant validation to ensure no loss of activity during shipping and storage. With national and international shipping restrictions of Tier 1 Select Agents and associated high costs, a universal BoNT standard presents a logistical and laboratory challenge. In addition, it has been demonstrated that several factors that are independent of a toxin standard can affect the outcome of the MBA [77,138,139], which has also been confirmed by years of experience in our laboratory. Such factors include the formulation of the toxin to be tested, diluents, treatment of the toxin sample prior to testing, consistency of the injection technique, and consistency in animal age, health, and housing.

### 5.3. Critical Aspects of Mouse Selection for the Detection of BoNTs

One critical factor that may affect the outcome of the MBA is the mouse strain used. A small study comparing two different BoNT/A formulations in three different mouse models showed toxin-specific and strain-specific differences, but statistical significance was not established [138]. The BAM manual does not specify a mouse strain, and to the best of our knowledge, no large-scale studies comparing sensitivities of different mouse strains to various BoNTs have been conducted. Considering the large number of BoNT types known by DNA sequencing, most BoNTs have not been isolated and comparatively evaluated for toxicity in the MBA. Notwithstanding the available data showing no mouse strain-specific differences, it is logical to use the same strain across laboratories. While inbred mouse strains would likely reduce the error of the MBA due to less biologic variation between mice, their increased cost makes their routine use not feasible for many laboratories with modest funding. A commonly available and widely used outbred mouse strain is CD1 (ICR) mice, which we have used successfully for many years in our laboratory. While there still may be biologic differences in CD1 mice bred in different breeding facilities, standardizing the MBA protocol to CD1 mice would reduce the chances for mouse strain-specific effects. Another critical factor is the age or weight range of the mice. The BAM manual suggests the use of mice of 16–24 g, and up to 34 g. This is a large weight range, and in addition, encompasses mice in various growth stages in their life cycle, as female CD1 mice weighing 16 g are on average three weeks old or younger, and mice weighing 34 g are about nine weeks old. Male CD1 mice are larger at a given age, such that male CD1 mice are about 18 g at three weeks, about 35 g at five weeks, and about 43 g at nine weeks of age. One small study analyzing a possible effect of small differences in mouse weight below 26 g on the outcome of the MBA for detection of BoNT/A,/B, or/C in culture supernatant or extract did not reveal statistically significant differences [140]. However, to the best of our knowledge, no controlled larger-scale studies have examined the relationship of BoNT sensitivity in mice at different developmental stages or weights using different BoNT preparations (including complex and purified BoNT). In our 30 years of experience conducting mouse bioassays in our laboratory, we have observed that larger/older mice appear to tolerate more BoNT than smaller/younger mice. While there are no data to our knowledge indicating that sex may be a variable in BoNT sensitivity, the weight-age differences of male and female mice means that male and female mice of the same weight may be at different developmental stages. In addition, if mice are pre-ordered from a vendor for MBAs and used within 2–4 weeks, their weight and maturity will change significantly during that time. Thus, standardizing the mouse strain, sex, and weight at the time of the MBA may aid in reducing the error associated with this assay.

### 5.4. Critical Aspects of Injection of BoNTs for the MBA

**Figure 2:**
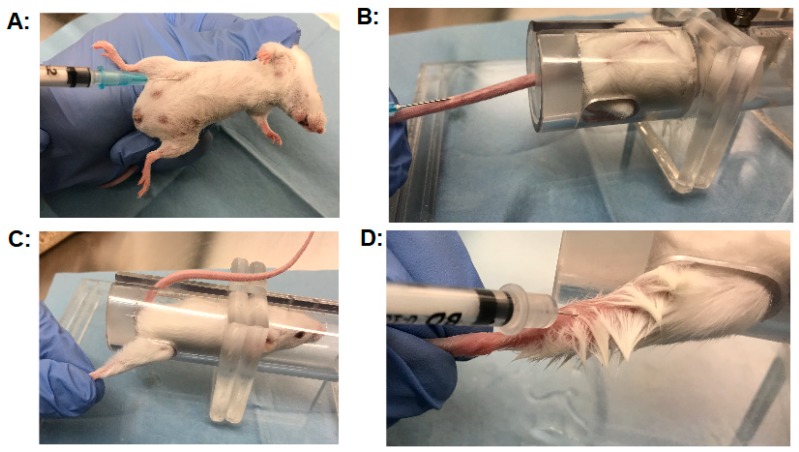
Mouse Injection techniques: (**A**) Intraperitoneal (IP) injections: The mouse is restraint in one hand by holding the scruff of the neck tightly between thumb and forefinger and the tail between pinky and palm of the hand and injected into the lower right quadrant of the peritoneum. (**B**) Intravenous (IV) injection: The mouse is restraint in a tube-like restraining device, with the tail being extended through a hole at the end of the tube. The tail vein is easily visible and is injected by inserting a needle into the vein at a shallow angle. (**C**) Restraint of a mouse for intramuscular injection into the right hind limb. (**D**) Intramuscular (IM) injection into the gastrocnemius muscle: The hind limb of the mouse is gently stretched, and the gastrocnemius muscle is injected with a small 30 gauge needle, inserting the needle to about themidpoint of the muscle. All animal experiments have been approved by the University of Wisconsin Institutional Animal Care and Use Committee (IACUC).

Another critical factor affecting the outcome of the MBA is the injection technique. Three injection techniques that are frequently used by our laboratory are discussed in detail in the following section. Guidelines for injection techniques are published [141,142] and are usually taught to laboratory staff by trained veterinarians. Nevertheless, errors can easily occur with the injection technique, and in our experience, it takes significant practice for new staff to master proper routine injections of BoNTs. For intraperitoneal injections, a volume of 0.5 mL is usually injected using a 1 mL syringe with a 25 gauge needle. Mice are restrained by firmly grasping the nape of the neck with the forefinger and thumb and holding the tail between the pinky finger in the palm of the hand, thereby exposing the ventral side of the animal (Figure 2a). The mouse should be firmly restrained without restricting breathing. The needle is then placed on the lower right quadrant of the animal using the dominant hand and gently inserted at an about 30–45° angle just into the peritoneum (Figure 2a). A gentle mild aspiration will ensure proper needle placement. Injection at a too steep angle or too close to the upper right quadrant could result in accidental injection and damage to the intestines or liver (bleeders). Injection of the BoNT solution should proceed at a moderate rate, with a second delay before gently retracting the needle. Injecting or retracting the needle to quickly can result in leakage of some of the material out of the injection site (leakers), causing inaccuracies in the assay. The injected mouse should be observed for a few seconds for potential leakage before placing it back in the cage, and any leakage should be recorded. For intravenous injection, mice are placed in a restraining device (Figure 2b), and the tail is gently yet firmly held straight back. The lateral tail vein is easily visible and is injected by inserting a 25 gauge needle at about the middle of the tail about 3 mm deep into the vein (with no aspiration, as this would lead to vein collapse), and the material is slowly and steadily injected. About 0.2 mL can be readily injected by this method, and our laboratory routinely uses 0.1 mL.

No resistance or swelling should be encountered, and the vein will visibly blanch with proper injection. Similar to intraperitoneal injection, the needle should be retracted slowly and any leakage recorded. For intramuscular injection into the right gastrocnemius muscle, mice are restrained in a device with an opening on the side through which the right hindlimb can be gently secured (Figure 2c). While holding the paw of the hindlimb, the needle is gently inserted into the top lateral section of the gastrocnemius muscle (Figure 2d), and the material is slowly injected. Only a volume up to 10 µl can be injected by this method, which can be accomplished with reasonable accuracy if using a 0.3 cc insulin syringe with 5μL markings and a 30 gauge needle or a Hamilton glass syringe. Since such a small volume is used for these injections, it is of particular importance to ensure that no leakage occurs from the injection site, and that the injected volume is consistent. Staff in our laboratory are usually trained by injecting increments of 10 µl onto a high precision scale until achieving an accuracy of ~ ±10%.

Irrespective of the injection technique, several toxin dilutions should always be used, and all mice should be counted in the results unless a mouse has been recorded for faulty injection (e.g., leakage). Some variability among outbred mice in response to BoNTs exists naturally and contributes to the variability in the results. In our laboratory with over 30 years of experience, all mice properly injected with four LD50 U of BoNT have always died. If some mice consistently show no symptoms at high toxin doses, it is most likely due to injection error, and the assay should be repeated. In addition, a statistically significant number of mice must be used to provide accuracy. Power analysis and our experience suggest that for a standard potency determination of a BoNT, six toxin dilutions in the range of 0.1–4 LD50 Units with four mice per group provides consistent and reliable data for research purposes.

### 5.5. Critical Aspects of Toxin Preparation for the MBA

Another critical factor in BoNT detection by the MBA is the diluent used to prepare BoNTs for injection. Use of GelPhos buffer (30 mM sodium phosphate (pH 6.3) and 0.2% gelatin) provides stability of BoNTs. Studies have shown that diluting BoNTs (from various pharmaceutical formulations) in saline resulted in significantly lower LD50 values than diluting the same samples in GelPhos buffer [136,138], and this is also corroborated by our laboratory experience. If BoNTs are contained in an acidic sample (pH < 4.5), such as a food sample that has spoiled throughout the study period, the toxin may precipitate out of solution and be removed during sample preparation by centrifugation. This could occur even if the sample is extracted in pH 6.3 GelPhos buffer if the acidity exceeds the buffering capacity of the GelPhos. In food challenge studies in our laboratory, we have encountered multiple occasions in which the food samples spoiled and acidified throughout the study period. The resulting GelPhos extracts had a pH of 3.0–4.5. When these samples were centrifuged to remove solid particles prior to pH adjustments, no toxicity was detected by the MBA. However, adjusting the pH prior to centrifugation resulted in mouse death within a few hours, indicating high levels of toxin. In addition, feeding an acidic preparation of BoNT to mice via oral gavage resulted in mouse death within the same time frame as pH-adjusted samples, indicating that the precipitated BoNTs are equally potent oral toxins as BoNTs in solution. Follow-up studies indicated that BoNT production occurred before obvious spoilage of the food product when the product may still be consumed, highlighting the importance of controlling for acid precipitation of BoNTs in food challenge studies.

Another important consideration for the MBA is that most BoNTs need to be proteolytically converted to the di-chain form for optimal biologic activity. BoNTs produced by proteolytic *C. botulinum* are usually present in the culture supernatant in the fully active di-chain form, while BoNTs produced by non-proteolytic *C. botulinum* or certain other BoNT-producing clostridia are not processed to the di-chain form and require activation by trypsin or LysC digestion prior to toxicity assessments. In addition, in young cultures of proteolytic *C. botulinum*, the BoNT may not yet be fully converted to the di-chain form, and BoNT extracted from cells that have not been released into the culture supernatant also may not be proteolytically activated. For accurate, specific activity determination of a BoNT, it is, therefore, important to first determine the amount of di-chain toxin by examination of reduced and non-reduced toxin by SDS-PAGE gel (Sodium Dodecyl Sulfate Polyacrylamide Gel Electrophoresis) or Western blot. If BoNT is not or only partially reduced to HC and LC, additional trypsin treatment is required. Trypsin has optimal activity at pH ~7.8–8.7 but activates botulinum toxins without significant degradation at pH 6–6.5. Thus, if trypsinization is required, it is essential that the pH of the sample is first adjusted to pH ~ 6.3. Trypsinization is usually conducted in GelPhos buffer at 35 °C for 30 min with 0.5% trypsin (Difco 1:250) [129], but it should be noted that trypsinization can also lead to BoNT degradation and trypsin conditions need to be optimized for each BoNT. Soybean trypsin inhibitor is routinely used in our laboratory after trypsinization for 30–60 min to prevent BoNT degradation during the following storage and handling.

When testing for BoNTs in various matrices, including many foods, false positives may result due to non-specific mouse deaths caused by the food samples. Several steps can be taken to avoid misinterpretation of results as deaths due to BoNTs. First, mice should be observed for symptoms indicating botulism within the first 24 h after injection, including ruffled fur, progressing paralysis, and difficulty breathing. The onset of symptoms varies depending on the toxin dose, but at least 30 min is usually required after BoNT injection for symptoms to be observed. The onset of symptoms is dose-dependent; thus, the injection of more dilute samples should result in a later onset of symptoms. If symptoms are observed within minutes after injection and dilution does not delay the onset of symptoms, they are likely caused by other food components. Coffee, chocolate, and certain fish extracts are typically substances that will result in significant visible discomfort of the mice immediately after injection. The BAM manual suggests heating a portion of a sample potentially containing BoNTs to 100 °C for 10 min to heat-inactivate the toxin as a negative control. However, other compounds that may cause non-specific deaths may also be heat-inactivated; thus, this step cannot unequivocally determine the cause of death. Deaths suspected to be due to botulism should be confirmed by performing a toxin neutralization assay [129], in which an excess of antitoxin is mixed with the BoNT-containing sample and mice are observed for prevention of symptoms and death.

### 5.6. Preparation of BoNT Samples for the MBA

There are many other factors during BoNT preparation that can affect the outcome of the MBA assay. These include inaccuracies of toxin dilutions, inactivation of the toxin due to deleterious handling and BoNT aggregation or oxidation, degradation of the BoNTs by food or clinical components, variations in purification or preparation methods of the BoNTs, purity of the BoNTs, the stability of the BoNTs in collected samples, excipients, and other factors. It is imperative to consider the physical and biological properties of the specific BoNT examined within the large and diverse family of BoNT proteins when conducting an MBA. For example, when examining a food sample for the presence of BoNTs, half of the sample should be trypsinized and half not trypsinized, to enable detection of BoNTs present in the di-chain form and BoNTs not yet activated and present in the 150 kDa single-chain. If the entire sample is trypsinized, BoNTs already present in the di-chain form may be degraded. Not trypsinizing could miss a BoNT that has not been converted to the di-chain or underestimate the quantity of the single chain BoNT. Similarly, when investigating a novel BoNT, the stability and percentage of di-chain and single-chain toxin has to be considered and should be determined prior to the MBA. Using a BoNT that is only partially converted to the di-chain form can lead to misleading results in studies such as antitoxin neutralization, as the single-chain BoNT will sometimes bind with greater affinity, yet the biologic activity is determined primarily by the di-chain form.

### 5.7. The MBA for BoNT Antitoxin and Inhibitor Detection

The MBA is frequently used to assess efficacy (neutralizing or inhibition) of an antitoxin or a putative inhibitor. While a non-lethal assay with a quantitative endpoint such as Rotarod or DAS (digit abduction score) score can be used at low and sub-lethal toxin doses, a time–to-death assay or quantal mouse neutralization assay should always use at least 10 LD50 U of toxin and numbers of animals for the statistical validity of the results. All control mice injected with 10 LD50 U of toxin should die. If some mice survive, the toxin dose, injection technique, dilution of toxin, excipient, and other factors must be considered for the optimization of the assay. If lower toxin doses are used, there’s a risk of some mice surviving due to natural variations of the tested population, which complicates interpretation of results and requires very large numbers of animals for statistically accurate interpretation. In our experience, all mice injected with >4 LD50 U die, but due to the intra-laboratory error of the MBA and absence of standardization of the assay, testing of a BoNT from another source and variations in assay conditions could lead to inadvertent administration of unintended biologic amounts (Units) of BoNT. Therefore, unless a parallel quantal MBA is conducted on the same toxin dilution used for the study, it is recommended that at least 10 LD50 U is used for inhibitor or antitoxin screens.

### 5.8. Time Required for the MBA

While the standard MBA suggests observation of the mice for four days [74,78], most mice will die within the first one or two days, depending on the BoNT type and dose. Symptoms in mice can appear as early as 30 min post-injection with very high doses, but the onset of symptoms can vary dramatically depending on the dose and BoNT type. The recent identification and characterization of the novel BoNT/FA have shown that some BoNT types can cause a significantly slower death, with mice starting to die at days two and three and continuing to die to day six [126,128,143]. Thus, mice should be observed at least daily until no more deaths are recorded, and mice with mild symptoms show signs of recovery.

Table 1 summarizes the most critical factors to consider for the quantal MBA and suggestions to achieve a more standardized assay across laboratories.

## 6. Conclusions

The MBA has been the gold standard BoNT detection assay for decades, yet the ethical concerns and cost associated with this assay have led to a strong push by regulatory agencies around the globe to reduce, refine, and replace this assay [144,145,146]. With careful consideration of the limitations and restriction of alternative assays, the use of the MBA can now be reduced significantly. However, it remains an essential tool in the characterization of the many BoNT types known today as well as novel BoNTs or putative BoNTs. It is the only assay that can examine the pharmacodynamic properties of BoNTs and the pathological or pharmaceutical effects after intoxication. The combination of this assay with human-specific (or other vertebrate-specific) cell models provides a powerful tool in expanding our understanding of BoNTs and their effects in humans, paving the way towards novel pharmaceutical applications as well as the developments of countermeasures. In addition, the MBA remains an important assay for BoNT detection in food or environmental samples, mostly due to the lack of an alternative method that is validated for all BoNTs.

NCB assays have the potential to largely replace the MBA for the purpose of potency determination of specific pharmaceutical BoNT formulations. NCBs can be easily standardized and detect only biologically active BoNTs if used at physiologically relevant doses, which is a requirement for potency determination of pharmaceutical BoNTs. In addition, NCB assays are excellent models for basic research of BoNTs, enabling cell entry studies and intracellular trafficking studies that would not be possible in vivo, as well as the screening of countermeasures. While decades of research efforts have focused on finding a cure for botulism that is effective after the toxin enters the neuronal cells, no compounds that can specifically enter neurons and inactivate the intracellular LC have been identified yet. Many effective inhibitors of catalytic LC activity have been identified, but most either do not enter neuronal cells efficiently, are not stable in cells, or cause toxicity. NCB assays are an excellent model to advance such studies without excessive use of animals. However, the examination of the potential metabolic conversion and pharmacokinetics of countermeasures will continue to require animal studies.

In summary, while the NCB assay and other in vitro assays now dramatically reduce the need to use the MBA for BoNT detection, the MBA remains an important mode of BoNT detection for many applications including basic research, analysis of pharmaceutical BoNTs to standardize and validate NCBs, detection in food and environmental samples, and detection and toxicity analyses of novel and putative BoNTs.

## Figures and Tables

**Table 1:** Suggestions for the standardized MBA procedure.

Critical Factor	Suggestion
Mouse strain	female CD1 (ICR)
Mouse weight	18–26 g at the time of the assay
caging	acclimate mice at least three days prior to assay, 4–5 mice per cage, 12 h dark-light cycle
Toxin diluent	GelPhos buffer (30mM sodium phosphate (pH 6.3) and 0.2% gelatin)
Injection volume	0.5 mL
Toxin preparation	ensure pH = 6.3 before centrifugation, examine toxin for proteolytic activation, trypsinize if needed
Toxin dilutions	Use several toxin dilutions. For quantal MBA, use a range from 0.1–4 LD50 U.
Injection technique	Careful slow injection in the lower right quadrant (IP), retract needle slowly, check for and record any leaks
For inhibitor/antitoxin studies	Use > 10 LD50 U
Time of assay	Until no mice have died for at least 24 h and mice are recovering from symptoms
Interpretation of results	All mice are counted, unless a bad injection was noted
Deaths	Confirm by antitoxin neutralization

## References

[1] Dressler D., Bhidayasiri R., Bohlega S., Chana P., Chien H.F., Chung T.M., Colosimo C., Ebke M., Fedoroff K., Frank B. (2018). Defining spasticity: A new approach considering current movement disorders terminology and botulinum toxin therapy. J. Neurol..

[2] Dressler D. (2016). Botulinum toxin drugs: Brief history and outlook. J. Neural Transm..

[3] Dressler D. (2012). Clinical applications of botulinum toxin. Curr. Opin. Microbiol..

[4] Webb R.P. (2018). Engineering of botulinum neurotoxins for biomedical applications. Toxins.

[5] Brown E.A., Schutz S.G., Simpson D.M. (2014). Botulinum toxin for neuropathic pain and spasticity: An overview. Pain Manag..

[6] Arnon S.S., Schechter R., Inglesby T.V., Henderson D.A., Bartlett J.G., Ascher M.S., Eitzen E., Fine A.D., Hauer J., Layton M. (2001). Botulinum toxin as a biological weapon: Medical and public health management. JAMA J. Am. Med. Assoc..

[7] Torii Y., Goto Y., Nakahira S., Kozaki S., Kaji R., Ginnaga A. (2014). Comparison of systemic toxicity between botulinum toxin subtypes A1 and A2 in mice and rats. Basic Clin. Pharm. Toxicol..

[8] Carli L., Montecucco C., Rossetto O. (2009). Assay of diffusion of different botulinum neurotoxin type a formulations injected in the mouse leg. Muscle Nerve.

[9] Cai B.B., Francis J., Brin M.F., Broide R.S. (2017). Botulinum neurotoxin type A-cleaved SNAP25 is confined to primary motor neurons and localized on the plasma membrane following intramuscular toxin injection. Neuroscience.

[10] Mazzocchio R., Caleo M. (2015). More than at the neuromuscular synapse: Actions of botulinum neurotoxin A in the central nervous system. Neuroscientist.

[11] Pellett S., Singh S.K. (2015). Pathogenesis of Clostridium botulinum in humans. Human Emerging and Re-Emerging Infections: Viral and Parasitic Infections, Volume I.

[12] Johnson E.A., Montecucco C., Engel A.G. (2008). Botulism. Handbook of Clinical Neurology.

[13] Dembek Z.F., Smith L.A., Rusnak J.M. (2007). Botulism: Cause, effects, diagnosis, clinical and laboratory identification, and treatment modalities. Disaster Med. Public Health Prep..

[14] Peck M.W., Smith T.J., Anniballi F., Austin J.W., Bano L., Bradshaw M., Cuervo P., Cheng L.W., Derman Y., Dorner B.G. (2017). Historical perspectives and guidelines for botulinum neurotoxin subtype nomenclature. Toxins.

[15] Pirazzini M., Rossetto O., Eleopra R., Montecucco C. (2017). Botulinum neurotoxins: Biology, pharmacology, and toxicology. Pharmacol. Rev..

[16] Montecucco C., Rasotto M.B. (2015). On botulinum neurotoxin variability. mBio.

[17] Mansfield M.J., Doxey A.C. (2018). Genomic insights into the evolution and ecology of botulinum neurotoxins. Pathog. Dis..

[18] Montal M. (2010). Botulinum neurotoxin: A marvel of protein design. Annu. Rev. Biochem..

[19] Lam K.H., Jin R. (2015). Architecture of the botulinum neurotoxin complex: A molecular machine for protection and delivery. Curr. Opin. Struct. Biol..

[20] Schiavo G., Rossetto O., Tonello F., Montecucco C. (1995). Intracellular targets and metalloprotease activity of tetanus and botulism neurotoxins. Curr Top Microbiol Immunol..

[21] Sudhof T.C., Rizo J. (2011). Synaptic vesicle exocytosis. Cold Spring Harb. Perspect. Biol..

[22] Sudhof T.C. (2004). The synaptic vesicle cycle. Annu. Rev. Neurosci..

[23] Rummel A. (2013). Double receptor anchorage of botulinum neurotoxins accounts for their exquisite neurospecificity. Curr. Top. Microbiol. Immunol..

[24] Rossetto O., Pirazzini M., Montecucco C. (2014). Botulinum neurotoxins: Genetic, structural and mechanistic insights. Nat. Rev. Microbiol..

[25] Montecucco C. (1986). How do tetanus and botulinum toxins bind to neuronal membranes?. Trends Biochem. Sci..

[26] Zanetti G., Azarnia Tehran D., Pirazzini M., Binz T., Shone C.C., Fillo S., Lista F., Rossetto O., Montecucco C. (2015). Inhibition of botulinum neurotoxins interchain disulfide bond reduction prevents the peripheral neuroparalysis of botulism. Biochem. Pharmacol..

[27] Pirazzini M., Azarnia Tehran D., Zanetti G., Lista F., Binz T., Shone C.C., Rossetto O., Montecucco C. (2015). The thioredoxin reductase--Thioredoxin redox system cleaves the interchain disulphide bond of botulinum neurotoxins on the cytosolic surface of synaptic vesicles. Toxicon.

[28] Pirazzini M., Azarnia Tehran D., Zanetti G., Megighian A., Scorzeto M., Fillo S., Shone C.C., Binz T., Rossetto O., Lista F. (2014). Thioredoxin and its reductase are present on synaptic vesicles, and their inhibition prevents the paralysis induced by botulinum neurotoxins. Cell Rep..

[29] Gimenez D.F., Gimenez J.A. (1995). The typing of botulinal neurotoxins. Int. J. Food Microbiol..

[30] Smith T.J., Lou J., Geren I.N., Forsyth C.M., Tsai R., Laporte S.L., Tepp W.H., Bradshaw M., Johnson E.A., Smith L.A. (2005). Sequence variation within botulinum neurotoxin serotypes impacts antibody binding and neutralization. Infect. Immun..

[31] Hill K.K., Xie G., Foley B.T., Smith T.J. (2015). Genetic diversity within the botulinum neurotoxin-producing bacteria and their neurotoxins. Toxicon.

[32] Zhang S., Masuyer G., Zhang J., Shen Y., Lundin D., Henriksson L., Miyashita S.I., Martinez-Carranza M., Dong M., Stenmark P. (2017). Identification and characterization of a novel botulinum neurotoxin. Nat. Commun..

[33] Hill K.K., Smith T.J., Helma C.H., Ticknor L.O., Foley B.T., Svensson R.T., Brown J.L., Johnson E.A., Smith L.A., Okinaka R.T. (2007). Genetic diversity among botulinum neurotoxin-producing clostridial strains. J. Bacteriol..

[34] Peck M.W. (2009). Biology and genomic analysis of Clostridium botulinum. Adv. Microb. Physiol..

[35] Stringer S.C., Carter A.T., Webb M.D., Wachnicka E., Crossman L.C., Sebaihia M., Peck M.W. (2013). Genomic and physiological variability within Group II (non-proteolytic) *Clostridium botulinum*. BMC Genom..

[36] Mansfield M.J., Wentz T.G., Zhang S., Lee E.J., Dong M., Sharma S.K., Doxey A.C. (2019). Bioinformatic discovery of a toxin family in *Chryseobacterium piperi* with sequence similarity to botulinum neurotoxins. Sci. Rep..

[37] Doxey A.C., Mansfield M.J., Montecucco C. (2018). Discovery of novel bacterial toxins by genomics and computational biology. Toxicon.

[38] Brunt J., Carter A.T., Stringer S.C., Peck M.W. (2018). Identification of a novel botulinum neurotoxin gene cluster in *Enterococcus*. FEBS Lett..

[39] Popoff M.R. (2018). Botulinum neurotoxins: Still a privilege of clostridia?. Cell Host Microbe.

[40] Zhang S., Lebreton F., Mansfield M.J., Miyashita S.I., Zhang J., Schwartzman J.A., Tao L., Masuyer G., Martinez-Carranza M., Stenmark P. (2018). Identification of a botulinum neurotoxin-like toxin in a commensal strain of *Enterococcus faecium*. Cell Host Microbe.

[41] Zornetta I., Azarnia Tehran D., Arrigoni G., Anniballi F., Bano L., Leka O., Zanotti G., Binz T., Montecucco C. (2016). The first non Clostridial botulinum-like toxin cleaves VAMP within the juxtamembrane domain. Sci. Rep..

[42] Mansfield M.J., Adams J.B., Doxey A.C. (2015). Botulinum neurotoxin homologs in non-*Clostridium* species. FEBS Lett..

[43] Swaminathan S. (2011). Molecular structures and functional relationships in clostridial neurotoxins. FEBS J..

[44] Fonfria E., Elliott M., Beard M., Chaddock J.A., Krupp J. (2018). Engineering botulinum toxins to improve and expand targeting and SNARE cleavage activity. Toxins.

[45] Dong M., Liu H., Tepp W.H., Johnson E.A., Janz R., Chapman E.R. (2008). Glycosylated SV2A and SV2B mediate the entry of botulinum neurotoxin E into neurons. Mol. Biol. Cell.

[46] Dong M., Yeh F., Tepp W.H., Dean C., Johnson E.A., Janz R., Chapman E.R. (2006). SV2 is the protein receptor for botulinum neurotoxin A. Science..

[47] Rummel A., Hafner K., Mahrhold S., Darashchonak N., Holt M., Jahn R., Beermann S., Karnath T., Bigalke H., Binz T. (2009). Botulinum neurotoxins C, E and F bind gangliosides via a conserved binding site prior to stimulation-dependent uptake with botulinum neurotoxin F utilising the three isoforms of SV2 as second receptor. J. Neurochem.

[48] Peng L., Tepp W.H., Johnson E.A., Dong M. (2011). Botulinum neurotoxin D uses synaptic vesicle protein SV2 and gangliosides as receptors. PLoS Pathog..

[49] Rummel A., Karnath T., Henke T., Bigalke H., Binz T. (2004). Synaptotagmins I and II act as nerve cell receptors for botulinum neurotoxin G. J. Biol. Chem..

[50] Dong M., Richards D.A., Goodnough M.C., Tepp W.H., Johnson E.A., Chapman E.R. (2003). Synaptotagmins I and II mediate entry of botulinum neurotoxin B into cells. J. Cell Biol..

[51] Nishiki T., Kamata Y., Nemoto Y., Omori A., Ito T., Takahashi M., Kozaki S. (1994). Identification of protein receptor for *Clostridium botulinum* type B neurotoxin in rat brain synaptosomes. J. Biol. Chem..

[52] Rummel A., Mahrhold S., Bigalke H., Binz T. (2011). Exchange of the H_CC_ domain mediating double receptor recognition improves the pharmacodynamic properties of botulinum neurotoxin. FEBS J..

[53] Kroken A.R., Blum F.C., Zuverink M., Barbieri J.T. (2017). Entry of botulinum neurotoxin subtypes A1 and A2 into neurons. Infect. Immun..

[54] Benoit R.M., Scharer M.A., Wieser M.M., Li X., Frey D., Kammerer R.A. (2017). Crystal structure of the BoNT/A2 receptor-binding domain in complex with the luminal domain of its neuronal receptor SV2C. Sci. Rep..

[55] Pier C.L., Chen C., Tepp W.H., Lin G., Janda K.D., Barbieri J.T., Pellett S., Johnson E.A. (2011). Botulinum neurotoxin subtype A2 enters neuronal cells faster than subtype A1. FEBS Lett..

[56] Keller J.E., Cai F., Neale E.A. (2004). Uptake of botulinum neurotoxin into cultured neurons. Biochemistry.

[57] Moritz M.S., Tepp W.H., Bradshaw M., Johnson E.A., Pellett S. (2018). Isolation and characterization of the novel botulinum neurotoxin A subtype 6. mSphere.

[58] Pellett S., Bradshaw M., Tepp W.H., Pier C.L., Whitemarsh R.C.M., Chen C., Barbieri J.T., Johnson E.A. (2018). The light chain defines the duration of action of botulinum toxin serotype A subtypes. MBio.

[59] Pellett S., Tepp W.H., Whitemarsh R.C., Bradshaw M., Johnson E.A. (2015). In vivo onset and duration of action varies for botulinum neurotoxin A subtypes 1–5. Toxicon.

[60] Whitemarsh R.C., Tepp W.H., Johnson E.A., Pellett S. (2014). Persistence of botulinum neurotoxin a subtypes 1-5 in primary rat spinal cord cells. PLoS ONE.

[61] Whitemarsh R.C., Tepp W.H., Bradshaw M., Lin G., Pier C.L., Scherf J.M., Johnson E.A., Pellett S. (2013). Characterization of botulinum neurotoxin A subtypes 1 through 5 by investigation of activities in mice, in neuronal cell cultures, and in vitro. Infect. Immun..

[62] Keller J.E. (2006). Recovery from botulinum neurotoxin poisoning in vivo. Neuroscience.

[63] Binz T., Blasi J., Yamasaki S., Baumeister A., Link E., Sudhof T.C., Jahn R., Niemann H. (1994). Proteolysis of SNAP-25 by types E and A botulinal neurotoxins. J. Biol. Chem..

[64] Blasi J., Chapman E.R., Link E., Binz T., Yamasaki S., De Camilli P., Sudhof T.C., Niemann H., Jahn R. (1993). Botulinum neurotoxin A selectively cleaves the synaptic protein SNAP-25. Nature.

[65] Schiavo G., Shone C.C., Bennett M.K., Scheller R.H., Montecucco C. (1995). Botulinum neurotoxin type C cleaves a single Lys-Ala bond within the carboxyl-terminal region of syntaxins. J. Biol. Chem..

[66] Blasi J., Chapman E.R., Yamasaki S., Binz T., Niemann H., Jahn R. (1993). Botulinum neurotoxin C1 blocks neurotransmitter release by means of cleaving HPC-1/syntaxin. EMBO J..

[67] Williamson L.C., Halpern J.L., Montecucco C., Brown J.E., Neale E.A. (1996). Clostridial neurotoxins and substrate proteolysis in intact neurons: Botulinum neurotoxin C acts on synaptosomal-associated protein of 25 kDa. J. Biol. Chem..

[68] Schiavo G., Malizio C., Trimble W.S., Polverino de Laureto P., Milan G., Sugiyama H., Johnson E.A., Montecucco C. (1994). Botulinum G neurotoxin cleaves VAMP/synaptobrevin at a single Ala-Ala peptide bond. J. Biol. Chem..

[69] Schiavo G., Shone C.C., Rossetto O., Alexander F.C., Montecucco C. (1993). Botulinum neurotoxin serotype F is a zinc endopeptidase specific for VAMP/synaptobrevin. J. Biol. Chem..

[70] Schiavo G., Benfenati F., Poulain B., Rossetto O., Polverino de Laureto P., DasGupta B.R., Montecucco C. (1992). Tetanus and botulinum-B neurotoxins block neurotransmitter release by proteolytic cleavage of synaptobrevin. Nature.

[71] Yamasaki S., Baumeister A., Binz T., Blasi J., Link E., Cornille F., Roques B., Fykse E.M., Sudhof T.C., Jahn R. (1994). Cleavage of members of the synaptobrevin/VAMP family by types D and F botulinal neurotoxins and tetanus toxin. J. Biol. Chem..

[72] Kalb S.R., Baudys J., Raphael B.H., Dykes J.K., Luquez C., Maslanka S.E., Barr J.R. (2015). Functional characterization of botulinum neurotoxin serotype H as a hybrid of known serotypes F and A (BoNT F/A). Anal. Chem..

[73] Kalb S.R., Baudys J., Webb R.P., Wright P., Smith T.J., Smith L.A., Fernandez R., Raphael B.H., Maslanka S.E., Pirkle J.L. (2012). Discovery of a novel enzymatic cleavage site for botulinum neurotoxin F5. FEBS Lett..

[74] Hatheway C.L., Balows A., Hausler W.H., Ohashi M., Turano M.A. (1988). Botulism. Laboratory Diagnosis of Infectious Diseases: Principles and Practice.

[75] Hatheway C.L. (1979). Laboratory procedures for cases of suspected infant botulism. Rev. Infect. Dis..

[76] Gimenez D.F., Ciccarelli A.S. (1970). Studies on strain 84 of Clostridium botulinum. Zentralblatt fur Bakteriologie Parasitenkunde Infektionskrankheiten und Hygiene Abt. I (Originale).

[77] Schantz E.J., Kautter D.A. (1978). Standardized assay for *Clostridium botulinum* toxins [in foods]. J. Assoc. Off. Anal. Chem..

[78] Wilder-Kofie T.D., Luquez C., Adler M., Dykes J.K., Coleman J.D., Maslanka S.E. (2011). An alternative in vivo method to refine the mouse bioassay for botulinum toxin detection. Comp. Med..

[79] Rasetti-Escargueil C., Liu Y., Rigsby P., Jones R.G., Sesardic D. (2011). Phrenic nerve-hemidiaphragm as a highly sensitive replacement assay for determination of functional botulinum toxin antibodies. Toxicon.

[80] Jones R.G., Alsop T.A., Hull R., Tierney R., Rigsby P., Holley J., Sesardic D. (2006). Botulinum type A toxin neutralisation by specific IgG and its fragments: A comparison of mouse systemic toxicity and local flaccid paralysis assays. Toxicon.

[81] Broide R.S., Rubino J., Nicholson G.S., Ardila M.C., Brown M.S., Aoki K.R., Francis J. (2013). The rat Digit Abduction Score (DAS) assay: A physiological model for assessing botulinum neurotoxin-induced skeletal muscle paralysis. Toxicon.

[82] Kutschenko A., Manig A., Reinert M.C., Monnich A., Liebetanz D. (2016). In-vivo comparison of the neurotoxic potencies of incobotulinumtoxinA, onabotulinumtoxinA, and abobotulinumtoxinA. Neurosci. Lett..

[83] Thirunavukkarasu N., Johnson E., Pillai S., Hodge D., Stanker L., Wentz T., Singh B., Venkateswaran K., McNutt P., Adler M. (2018). Botulinum neurotoxin detection methods for public health response and surveillance. Front. Bioeng. Biotechnol..

[84] Simon S., Fiebig U., Liu Y., Tierney R., Dano J., Worbs S., Endermann T., Nevers M.C., Volland H., Sesardic D. (2015). Recommended immunological strategies to screen for botulinum neurotoxin-containing samples. Toxins.

[85] Kalb S.R., Baudys J., Wang D., Barr J.R. (2015). Recommended mass spectrometry-based strategies to identify botulinum neurotoxin-containing samples. Toxins.

[86] Kalb S.R., Boyer A.E., Barr J.R. (2015). Mass spectrometric detection of bacterial protein toxins and their enzymatic activity. Toxins.

[87] Worbs S., Fiebig U., Zeleny R., Schimmel H., Rummel A., Luginbuhl W., Dorner B.G. (2015). Qualitative and quantitative detection of botulinum neurotoxins from complex matrices: Results of the first international proficiency test. Toxins.

[88] Dorner M.B., Schulz K.M., Kull S., Dorner B.G. (2013). Complexity of botulinum neurotoxins: Challenges for detection technology. Curr. Top. Microbiol. Immunol..

[89] Pellett S. (2013). Progress in cell based assays for botulinum neurotoxin detection. Curr. Top. Microbiol. Immunol..

[90] Kalb S.R., Baudys J., Kiernan K., Wang D., Becher F., Barr J.R. (2019). Proposed BoNT/A and/B peptide substrates cannot detect multiple subtypes in the Endopep-MS assay. J. Anal. Toxicol..

[91] Hobbs R.J., Thomas C.A., Halliwell J., Gwenin C.D. (2019). Rapid detection of botulinum neurotoxins—A Review. Toxins.

[92] Wheeler C., Inami G., Mohle-Boetani J., Vugia D. (2009). Sensitivity of mouse bioassay in clinical wound botulism. Clin. Infect. Dis..

[93] Halai U.A., Terashita D., Kim M., Green N., Kalb S.R., Chatham-Stephens K., Balter S. (2018). Notes from the field: Intestinal colonization and possible iatrogenic botulism in mouse bioassay-negative serum specimens—Los Angeles County, California, November 2017. MMWR Morb. Mortal. Wkly. Rep..

[94] Rowlands R.E., Ristori C.A., Lopes G.I., Paula A.M., Sakuma H., Grigaliunas R., Lopreato Filho R., Gelli D.S., Eduardo M.B., Jakabi M. (2010). Botulism in Brazil, 2000–2008: Epidemiology, clinical findings and laboratorial diagnosis. Revista do Instituto de Medicina Tropical de São Paulo.

[95] Shahcheraghi F., Nobari S., Masoumi Asl H., Aslani M.M. (2013). Identification of botulinum toxin type in clinical samples and foods in Iran. Arch. Iran. Med..

[96] Fernandez-Salas E., Wang J., Molina Y., Nelson J.B., Jacky B.P., Aoki K.R. (2012). Botulinum neurotoxin serotype A specific cell-based potency assay to replace the mouse bioassay. PLoS ONE.

[97] Mander G., Bruenn C., Jatzke C., Eisele K.H., Taylor H.V., Pellett S., Johnson E.A., Fink K. (2015). Potency assay for botulinum neurotoxin type A based on neuronal cells as a replacement for the mouse bioassay. Toxicon.

[98] Whitemarsh R.C., Strathman M.J., Chase L.G., Stankewicz C., Tepp W.H., Johnson E.A., Pellett S. (2012). Novel application of human neurons derived from induced pluripotent stem cells for highly sensitive botulinum neurotoxin detection. Toxicol. Sci..

[99] Pathe-Neuschafer-Rube A., Neuschafer-Rube F., Haas G., Langoth-Fehringer N., Puschel G.P. (2018). Cell-based reporter release assay to determine the potency of proteolytic bacterial neurotoxins. Toxins.

[100] Jenkinson S.P., Grandgirard D., Heidemann M., Tscherter A., Avondet M.A., Leib S.L. (2017). Embryonic stem cell-derived neurons grown on multi-electrode arrays as a novel in vitro bioassay for the detection of *Clostridium botulinum* neurotoxins. Front. Pharm..

[101] Torgeman A., Diamant E., Levin L., David A.B., Epstein E., Girshengorn M., Mazor O., Rosenfeld R., Zichel R. (2017). An in vitro cell-based potency assay for pharmaceutical type A botulinum antitoxins. Vaccine.

[102] Pellett S., Du Z.W., Pier C.L., Tepp W.H., Zhang S.C., Johnson E.A. (2011). Sensitive and quantitative detection of botulinum neurotoxin in neurons derived from mouse embryonic stem cells. Biochem. Biophys. Res. Commun..

[103] Pellett S., Tepp W.H., Toth S.I., Johnson E.A. (2010). Comparison of the primary rat spinal cord cell (RSC) assay and the mouse bioassay for botulinum neurotoxin type A potency determination. J. Pharmacol. Toxicol. Methods.

[104] Pellett S., Tepp W.H., Clancy C.M., Borodic G.E., Johnson E.A. (2007). A neuronal cell-based botulinum neurotoxin assay for highly sensitive and specific detection of neutralizing serum antibodies. FEBS Lett..

[105] Hubbard K.S., Gut I.M., Lyman M.E., Tuznik K.M., Mesngon M.T., McNutt P.M. (2012). High yield derivation of enriched glutamatergic neurons from suspension-cultured mouse ESCs for neurotoxicology research. BMC Neuroscience.

[106] McNutt P., Celver J., Hamilton T., Mesngon M. (2011). Embryonic stem cell-derived neurons are a novel, highly sensitive tissue culture platform for botulinum research. Biochem. Biophys. Res. Commun..

[107] Kiris E., Kota K.P., Burnett J.C., Soloveva V., Kane C.D., Bavari S. (2014). Recent developments in cell-based assays and stem cell technologies for botulinum neurotoxin research and drug discovery. Expert Rev. Mol. Diagn..

[108] Kiris E., Nuss J.E., Burnett J.C., Kota K.P., Koh D.C., Wanner L.M., Torres-Melendez E., Gussio R., Tessarollo L., Bavari S. (2011). Embryonic stem cell-derived motoneurons provide a highly sensitive cell culture model for botulinum neurotoxin studies, with implications for high-throughput drug discovery. Stem Cell Res..

[109] Nuss J.E., Ruthel G., Tressler L.E., Wanner L.M., Torres-Melendez E., Hale M.L., Bavari S. (2010). Development of cell-based assays to measure botulinum neurotoxin serotype A activity using cleavage-sensitive antibodies. J. Biomol. Screen..

[110] Pellett S., Tepp W.H., Johnson E.A. (2019). Botulinum neurotoxins A, B, C, E, and F preferentially enter cultured human motor neurons compared to other cultured human neuronal populations. FEBS Lett..

[111] Duarte G.S., Castelao M., Rodrigues F.B., Marques R.E., Ferreira J., Sampaio C., Moore A.P., Costa J. (2016). Botulinum toxin type A versus botulinum toxin type B for cervical dystonia. Cochrane Database Syst. Rev..

[112] Blitzer A. (2005). Botulinum toxin A and B: A comparative dosing study for spasmodic dysphonia. Otolaryngol. Head Neck Surg..

[113] Flynn T.C. (2004). Myobloc. Dermatol. Clin..

[114] Yu J., Thomson J.A. (2008). Pluripotent stem cell lines. Genes Dev..

[115] Takahashi K., Tanabe K., Ohnuki M., Narita M., Ichisaka T., Tomoda K., Yamanaka S. (2007). Induction of pluripotent stem cells from adult human fibroblasts by defined factors. Cell.

[116] Yu J., Vodyanik M.A., Smuga-Otto K., Antosiewicz-Bourget J., Frane J.L., Tian S., Nie J., Jonsdottir G.A., Ruotti V., Stewart R. (2007). Induced pluripotent stem cell lines derived from human somatic cells. Science..

[117] Gordon J., Amini S., White M.K. (2013). General overview of neuronal cell culture. Methods Mol. Biol..

[118] Simpson L. (2013). The life history of a botulinum toxin molecule. Toxicon.

[119] Ravichandran E., Gong Y., Al Saleem F.H., Ancharski D.M., Joshi S.G., Simpson L.L. (2006). An initial assessment of the systemic pharmacokinetics of botulinum toxin. J. Pharmacol. Exp. Ther..

[120] Al-Saleem F.H., Ancharski D.M., Ravichandran E., Joshi S.G., Singh A.K., Gong Y., Simpson L.L. (2008). The role of systemic handling in the pathophysiologic actions of botulinum toxin. J. Pharmacol. Exp. Ther..

[121] Black J.D., Dolly J.O. (1986). Interaction of 125I-labeled botulinum neurotoxins with nerve terminals. I. Ultrastructural autoradiographic localization and quantitation of distinct membrane acceptors for types A and B on motor nerves. J. Cell Biol..

[122] Black J.D., Dolly J.O. (1987). Selective location of acceptors for botulinum neurotoxin A in the central and peripheral nervous systems. Neuroscience.

[123] Schiavo G., Matteoli M., Montecucco C. (2000). Neurotoxins affecting neuroexocytosis. Physiol. Rev..

[124] Montecucco C., Schiavo G. (1994). Mechanism of action of tetanus and botulinum neurotoxins. Mol. Microbiol..

[125] Bomba-Warczak E., Vevea J.D., Brittain J.M., Figueroa-Bernier A., Tepp W.H., Johnson E.A., Yeh F.L., Chapman E.R. (2016). Interneuronal transfer and distal action of tetanus toxin and botulinum neurotoxins A and D in central neurons. Cell Rep..

[126] Pellett S., Tepp W.H., Lin G., Johnson E.A. (2018). Substrate cleavage and duration of action of botulinum neurotoxin type FA (“H, HA”). Toxicon.

[127] Pellett S., Tepp W.H., Bradshaw M., Kalb S.R., Dykes J.K., Lin G., Nawrocki E.M., Pier C.L., Barr J.R., Maslanka S.E. (2016). Purification and characterization of botulinum neurotoxin FA from a genetically modified *Clostridium botulinum* strain. mSphere.

[128] Maslanka S.E., Luquez C., Dykes J.K., Tepp W.H., Pier C.L., Pellett S., Raphael B.H., Kalb S.R., Barr J.R., Rao A. (2016). A novel botulinum neurotoxin, previously reported as serotype H, has a hybrid-like structure with regions of similarity to the structures of serotypes A and F and is neutralized with serotype A antitoxin. J. Infect. Dis..

[129] Clostridium Botulinum. https://www.fsis.usda.gov/wps/portal/fsis/topics/food-safety-education/get-answers/food-safety-fact-sheets/foodborne-illness-and-disease/clostridium-botulinum/ct_index.

[130] Kondo H., Shimizu T., Kubonoya M., Izumi N., Takahashi M., Sakaguchi G. (1984). Titration of botulinum toxins for lethal toxicity by intravenous injection into mice. Jpn. J. Med. Sci. Biol..

[131] Boroff D.A., Fleck U. (1966). Statistical analysis of a rapid in vivo method for the titration of the toxin of Clostridium botulinum. J. Bacteriol..

[132] Lamanna C., Spero L., Schantz E.J. (1970). Dependence of time to death on molecular size of botulinum toxin. Infect. Immun..

[133] Pellett S., Tepp W.H., Scherf J.M., Pier C.L., Johnson E.A. (2015). Activity of botulinum neurotoxin type D (strain 1873) in human neurons. Toxicon.

[134] Peng L., Berntsson R.P., Tepp W.H., Pitkin R.M., Johnson E.A., Stenmark P., Dong M. (2012). Botulinum neurotoxin D-C uses synaptotagmin I/II as receptors and human synaptotagmin II is not an effective receptor for type B, D-C, and G toxins. J. Cell Sci..

[135] Strotmeier J., Willjes G., Binz T., Rummel A. (2012). Human synaptotagmin-II is not a high affinity receptor for botulinum neurotoxin B and G: Increased therapeutic dosage and immunogenicity. FEBS Lett..

[136] Sesardic D., Leung T., Gaines Das R. (2003). Role for standards in assays of botulinum toxins: International collaborative study of three preparations of botulinum type A toxin. Biologicals.

[137] Weisemann J., Krez N., Fiebig U., Worbs S., Skiba M., Endermann T., Dorner M.B., Bergstrom T., Munoz A., Zegers I. (2015). Generation and characterization of six recombinant botulinum neurotoxins as reference material to serve in an international proficiency test. Toxins.

[138] McLellan K., Das R.E., Ekong T.A., Sesardic D. (1996). Therapeutic botulinum type A toxin: Factors affecting potency. Toxicon.

[139] Woodburn M.J., Somers E., Rodriguez J., Schantz E.J. (1979). Heat inactivation rates of botulinum toxins A, B, E and F in some foods and buffers. J. Food Sci..

[140] Lamanna C., Jensen W.I., Bross I.D. (1955). Body weight as a factor in the response of mice to botulinal toxins. Am. J. Hyg..

[141] Shimizu S., Hedrich H.J., Bullock G. (2004). Routes of administration. The Laboratory Mouse.

[142] US National Institutes of Health Basic Biomethodology for Laboratory Mice. https://theodora.com/rodent_laboratory/injections.html.

[143] Barash J.R., Arnon S.S. (2014). A novel strain of *Clostridium botulinum* that produces type B and type H botulinum toxins. J. Infect. Dis..

[144] Sesardic D. Approaches to replace mouse LD50 assay for botulinum toxins. Proceedings of the Toxins.

[145] Adler S., Bicker G., Bigalke H., Bishop C., Blumel J., Dressler D., Fitzgerald J., Gessler F., Heuschen H., Kegel B. (2010). The current scientific and legal status of alternative methods to the LD_50_ test for botulinum neurotoxin potency testing. The report and recommendations of a ZEBET Expert Meeting. Altern. Lab. Anim..

[146] Sesardic D., Gaines Das R. (2007). Alternatives to the LD_50_ assay for botulinum toxin potency testing: Strategies and progress towards refinement, reduction and replacement. AATEX.

